# Influence of Atg5 Mutation in SLE Depends on Functional IL-10 Genotype

**DOI:** 10.1371/journal.pone.0078756

**Published:** 2013-10-18

**Authors:** Patricia López, Elisa Alonso-Pérez, Javier Rodríguez-Carrio, Ana Suárez

**Affiliations:** 1 Department of Functional Biology, Immunology Area, Faculty of Medicine, University of Oviedo, Oviedo, Spain; 2 Laboratorio de Investigación, Instituto de Investigación Sanitaria, Hospital Clínico Universitario de Santiago, Santiago de Compostela, Spain; University of Texas Health Science Center at Houston, United States of America

## Abstract

Increasing evidence supports the involvement of autophagy in the etiopathology of autoimmune diseases. Despite the identification of autophagy-related protein (Atg)-5 as one of the susceptibility loci in systemic Lupus erythematosus (SLE), the consequences of the carriage of these mutations for patients remain unclear. The present work analyzed the association of Atg5 rs573775 single nucleotide polymorphism (SNP) with SLE susceptibility, IFNα, TNFα and IL-10 serum levels, and clinical features, in 115 patients and 170 healthy individuals. Patients who where carriers of the rs573775 T* minor allele presented lower IFNα levels than those with the wild genotype, whereas the opposite result was detected for IL-10. Thus, since IL-10 production was regulated by rs1800896 polymorphisms, we evaluated the effect of this Atg5 mutation in genetically high and low IL-10 producers. Interestingly, we found that the rs573775 T* allele was a risk factor for SLE in carriers of the high IL-10 producer genotype, but not among genetically low producers. Moreover, IL-10 genotype influences SLE features in patients presenting the Atg5 mutated allele. Specifically, carriage of the rs573775 T* allele led to IL-10 upregulation, reduced IFNα and TNFα production and a low frequency of cytopenia in patients with the high IL-10 producer genotype, whereas patients with the same Atg5 allele that were low IL-10 producers presented reduced amounts of all these cytokines, had a lower prevalence of anti-dsDNA antibodies and the latest onset age. In conclusion, the Atg5 rs573775 T* allele seems to influence SLE susceptibility, cytokine production and disease features depending on other factors such as functional IL-10 genotype.

## Introduction

Systemic lupus erythematosus (SLE) is a multifactorial autoimmune disease characterized by abnormal B and T cell activation, cytokine dysregulation and production of autoantibodies against cellular components generated from dying cells [1]. The deposit of the resulting immune-complexes can cause tissue injury in multiple organs, including skin, muscle, joints, kidneys and heart [2]. Moreover, immune-complexes containing autoantibodies against DNA and ribonucleoproteins may activate the Toll-like receptors (TLR)-7 and 9 in many cell types, including monocytes, dendritic cells (DC) and B lymphocytes, thus increasing the transcription of multiple proinflammatory mediators and type I interferons (IFN). In fact, IFNα secretion by plasmacytoid DC (pDC) plays a key role in the pathology of the disease [2,3]. 

A number of cellular abnormalities have been observed in SLE patients. Among them, the increased apoptosis rate and the impaired clearance of apoptotic bodies [4,5] may cause harmful effects, since they provide a source of autoantigens to elicit the autoimmune response [6]. Whit this in mind, the autophagy pathway plays a protective role, delivering signals for the clearance of apoptotic cells as well as genomic stability [7,8]. Autophagy is a cellular recycling process for damaged organelles/proteins that were previously sequestered in autophagosomes, double-membrane vacuoles whose formation is regulated by several autophagy-related gene (Atg) proteins, and then degraded after fusion with lysosomes [9,10]. In addition, autophagy participates in relevant immunological functions, including pathogen elimination, antigen presentation, lymphocyte development and inflammatory regulation [10–12]. In line with this, it has been reported that Atg5 contributes to the clearance of apoptotic bodies [7], influences DC antigen presentation [13] and may regulate the secretion of pro-inflammatory cytokines [14,15].

Accumulating evidence indicates that aberrant regulation of autophagy-dependent functions could be a key component of the etiology of autoimmune diseases, such as SLE [16,17]. In this regard, a dysregulation of autophagy has been described in T cells from lupus-prone mice and in patients with SLE [18]. In addition, serum factors, likely autoantibodies, purified from patients with active SLE have been shown to induce autophagy in neuroblastoma cell lines, providing a link between autophagy and SLE [19]. 

More recently, genetic studies reported that mutations in various autophagy regulators may contribute to the pathogenesis of SLE [20]. In this context, genome-wide association studies in different populations have linked several single nucleotide polymorphisms (SNPs) in Atg5 to SLE susceptibility [21–24]. Although the functional effects of these Atg5 SNPs are yet unknown, it has been reported that altered expression of this molecule generates autoimmunity and multi-organ inflammation in mice [25]. Nevertheless, despite the suggested role of autophagy in SLE pathogenesis, the consequences of the presence of Atg5 mutations for SLE patients remain unclear. Thus, in the present work we have analyzed the possible association of Atg5 rs573775 SNP, related to SLE susceptibility in large cohorts [21,26], with serum levels of IFNα, TNFα and IL-10, pathogenic cytokines for SLE that were usually upregulated in patients. In addition, influence on disease susceptibility and clinical characteristics of SLE patients were also evaluated.

## Materials and Methods

### Ethics statement

Ethics approval for this study was obtained from the Regional Ethics Committee for Clinical Research (Servicio de Salud del Principado de Asturias), according to the Declaration of Helsinki. Written informed consent was signed from all individuals prior to participation in the study.

### SLE patients

All patients included in the study (n=115) were recruited from the Asturian SLE Register [27,28], were all white in origin, and fulfilled the American College of Rheumatology (ACR) criteria for SLE [29]. Information on clinical features during the disease course was obtained after a detailed review of clinical histories (Table 1). At the time of sampling no patient had flares of disease activity and they were asked precise questions regarding the treatment received over the previous 3 months. Sex and age-matched healthy controls (n=170) were enlisted from the Asturian Blood Transfusion Center.

**Table 1:** Demographic and clinical features of SLE patients.

**Total SLE patients**	**115**
Sex (female/male) (n)	108/7
Age at diagnosis, years (mean±SEM)	30.39 ± 1.15
Disease duration, years (mean±SEM)	12.48 ± 0.79
Clinical manifestations, n (%)	
Malar rash	68 (59.1)
Discoid lesions	23 (20.0)
Photosensitivity	65 (56.5)
Oral ulcers	46 (40.0)
Arthritis	86 (74.8)
Serositis	25 (21.7)
Cytopenia	61 (53.0)
Renal disorder	36 (31.6)
Neurological disorder	8 (7.0)
Autoantibodies, n (%)	
ANAs	113 (98.3)
Anti-dsDNA / titer, U/ml (mean±SEM)	85 (73.9)/34.19 ± 7.09
Anti-SSa	38 (33.0)
Anti-SSb	17 (14.8)
Anti-Sm	9 (7.8)
Anti-RNP	17 (14.8)
RF	23 (20.0)
Treatment, n (%)	
None or NSAIDs	19 (16.5)
Antimalarial drugs	64 (55.7)
Glucocorticoids/Dose, mg/day (mean±SEM)	63 (54.8)/7.80 ± 0.99)
Immunosuppressive drugs^a^	18 (15.7)

^a^ Methotrexate, azathioprine, cyclophosphamide, cyclosporine A or mycophenolatemophetil.

dsDNA: double stranded DNA; RF: Rheumatoid factor; NSAID: non-steroidal anti-inflammatory drug.

### Atg5 and IL-10 genotyping

DNA was obtained from peripheral blood cells of 115 SLE patients and 170 healthy controls by standard procedures. SNP rs1800896 (A/G) at position -1082 on the IL-10 gene was determined after amplification and hybridization with fluorescent-labeled probes (LightCycler, Roche Diagnostics, Mannheim, Germany), as previously described [30]. The primers used were: 5’-atccaagacaacactactaaggc and 5’-atggggtggaagaagttgaa, and the hybridization probes were ggataggaggtcccttactttcctcttacc-F and LC Red 640-ccctacttccccctcccaaa. 

To determine the intronic SNP rs573775 (C/T) located in Atg5 gene, DNA was amplified, then products were purified by Exo-SAP digestion with exonuclease I (Epicentre, Madison, WI, USA) and shrimp alkaline phosphatase (GE Healthcare, Barcelona, Spain) and next single-base extension reactions were performed with the SNaPshot Multiplex kit (Applied Biosystems, Foster City, CA, USA). Samples were analyzed in an AbiPrism 3130xl Genetic Analyzer (Applied Biosystems). The primers used were: 5’-agaggtcaaaagccatgtcc and 5’-cccctgaacacctttctctg; and the probe was 5´-TTACCTATGATTGATCGTGGTGActcacagc**T**ctctggcccca. This probe was extended with a 5' tail (in capitals) that has no homology with human sequences and mutated (in bold and capital) to avoid dimer formation.

### Cytokine quantification

Serum samples were collected and maintained at -80°C until cytokine determination. IFNα levels were quantified by ELISA (PBL Biomedical, USA) following the manufacturer’s instructions. TNFα and IL-10 levels were determined by an in-house ELISA as previously described [31,32]. Briefly, microtiter wells were coated overnight with affinity purified anti-human TNFα or anti-human IL-10 monoclonal antibody (R&D Systems, Abingdon, UK) and blocked with 1% casein in Tris Buffered Saline (TBS) for two hours at 37°C. Serum samples and TNFα/IL-10 standards (R&D), diluted in blocking solution, were then incubated for 18 hours at 4°C. After washing with TBS/Tween 20 (0.05%), wells were incubated for two hours with biotinylated antihuman TNFα/IL-10 monoclonal antibody (R&D), washed, incubated for one hour with streptavidin-alkaline phosphatase conjugate and revealed using *p*-nitrophenyl phosphate as substrate. Absorbance was determined at a wavelength of 405 nm. Quantities of both cytokines were calculated according to the corresponding standard curves. The lower limit of detection was 3.12 pg/ml for IFNα and 7.5 pg/ml for TNFα and IL-10. 

### Statistical analysis

The Kolmogorov-Smirnov test was used to assess the normal distribution of the data. Data of cytokine serum levels were represented by median (interquartile range) and differences between patients and controls were assessed by the non-parametric Mann-Whitney U-test or the Kruskal-Wallis test. Genotype frequencies were obtained by direct counting and their distribution between SLE patients and controls was compared using 3 × 2 contingency tables and the χ^2^ test. Association of Atg5 rs573775, IL-10 rs1800896 SNPs and their interaction on SLE susceptibility was calculated by logistic regression modeling adjusted for age and gender (Wald test). Multivariate linear regression analyses were performed to determine the influence of IL-10 and Atg5 SNPs on cytokine serum levels in SLE patients. Models were adjusted for age and sex and standardized linear regression coefficients (beta) were used as an estimate of the association. The possible effect of rs573775 - rs1800896 interaction on cytokine serum levels were evaluated by multivariate regression analyses. The differences in clinical features of SLE patients on basis of IL-10/Atg5 combined genotype were evaluated by the χ^2^ test for categorical variables or the Mann Whitney U test for continuous variables. GraphPad Prism 5 software (GraphPad Software), SPSS 19.0 software package (SPSS Inc) and R package 2.4.1. (www.r-project.org) were used for all determinations and a p<0.05 was considered significant.

## Results

### 1. IFNα and IL-10 levels were associated with Atg5 genotype in SLE patients

It has been reported that mutations in the Atg5 gene could be a risk factor for SLE. Thus, we analyzed the possible association of the SNP rs573775 (C>T) with serum levels of IFNα, IL-10 and TNFα, three relevant cytokines usually increased in this disease. Thus, 170 healthy controls and 115 SLE patients were genotyped and cytokine levels determined in serum samples. Table 1 shows demographic and clinical characteristics of patients included in the study. 

**Figure 1:**
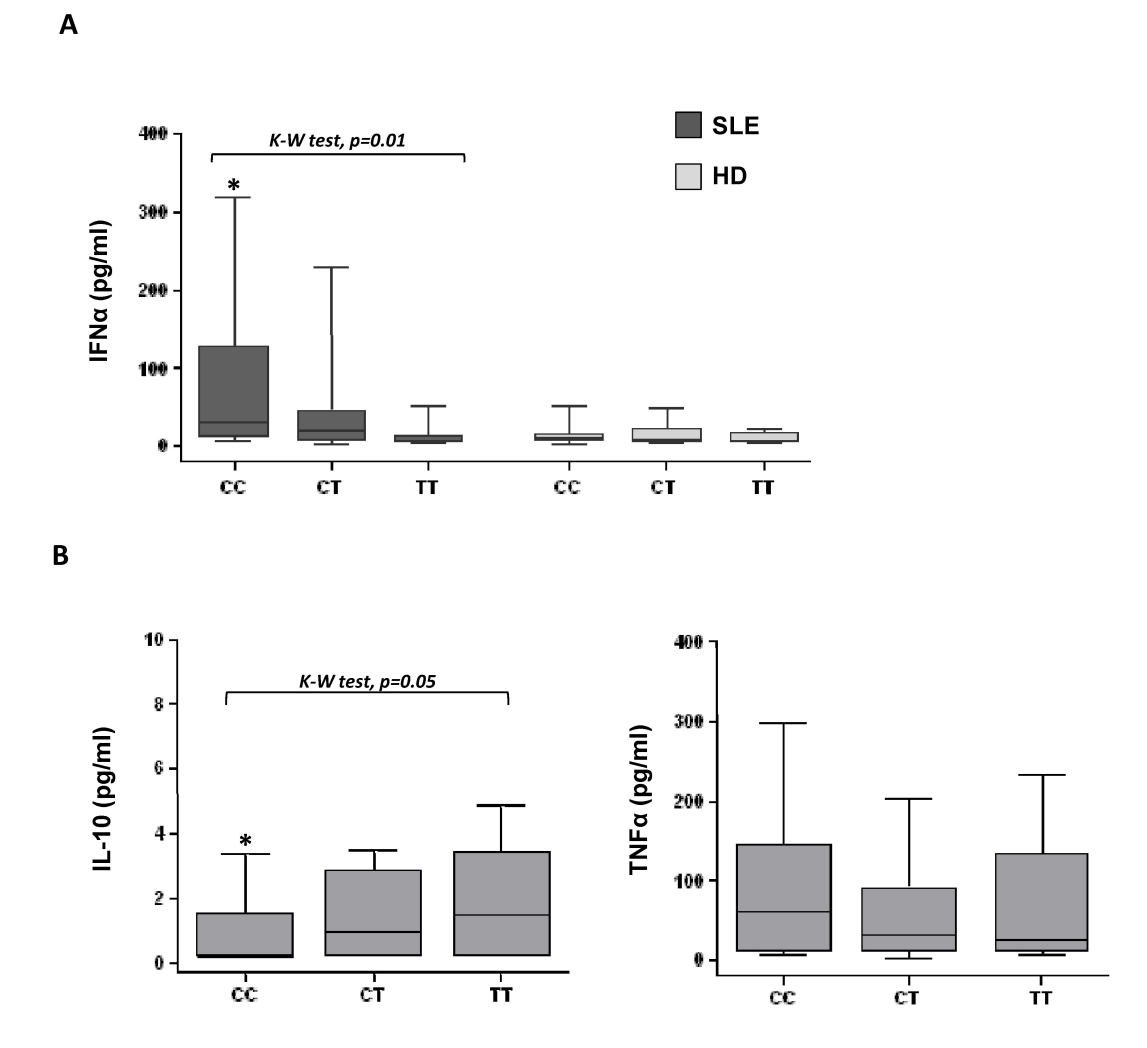
Atg5 rs573775 SNP influences cytokine levels in SLE patients. IFNα, TNFα and IL-10 serum levels were quantified by ELISA techniques in individuals previously genotyped for rs573775 SNP. A) IFNα serum levels in SLE patients and controls are shown on the basis of the Atg5 rs573775 alleles. B) IL-10 and TNFα serum levels depending on Atg5 alleles in SLE patients. Data are shown as box plots, where the lines in the boxes represent the median, the boxes represent the 25^th^ to 75^th^ centiles, and the lines outside the boxes represent the 10^th^ and 90^th^ centiles. Differences in cytokine serum concentration among genotypes were analyzed by the Kruskal-Wallis test (K-W test). Significant differences between one group and the rest were evaluated by Mann-Whitney U test (*p<0.05).

 Serum levels of IFNα and TNFα were significantly higher in patients compared with controls [IFNα: 19.61 (44.61) *vs* 9.91 (11.52), p<0.001; TNFα: 41.26 (113.56) *vs* 18.83 (49.52), p=0.02], whereas IL-10 were detected in SLE patients [0.23 (2.84)] but not in most healthy individuals. Grouping by rs573775 SNP did not affect cytokine levels in controls, but significant differences were detected in SLE patients. Figure 1 shows that presence of the Atg5 T* minor allele was associated with lower production of IFNα (median values, CC: 31.95, CT: 18.79, TT: 6.42, pg/ml; p=0.01, Kruskal-Wallis test), while TNFα levels, which were positively correlated with IFNα in patients (r=0.280, p<0.01), showed a similar but not significant trend (median values, CC: 61.19, CT: 31.73, TT: 24.99 pg/ml). Conversely, an opposite effect was observed for IL-10, Atg5 T* allele carrier patients presenting the highest serum levels (median values, CC: 0.20, CT: 0.95, TT: 1.49, pg/ml; p=0.05, Kruskal-Wallis test). 

Thus, our data showed an unexpected association between presence of the rs573775 T* mutated allele and low ability to upregulate IFNα levels. On the contrary, this allele was related to high amounts of IL-10, a cytokine genetically regulated by known functional promoter polymorphisms that have been previously associated with SLE susceptibility and/or outcome in several populations [33].

### 2. The effect of Atg5 alleles on SLE susceptibility was influenced by IL-10 genotype

In view of the relationship between presence of the Atg5 T* mutated allele and high IL-10 serum levels in SLE patients, and given the reported association of the IL-10 rs1800896 G* allele with high IL-10 production [34], we wanted to evaluate the possible effect of both Atg5 and IL-10 SNPs, alone or in combination, on SLE susceptibility. Initial analysis of the genotypic and allelic frequencies of Atg5 and IL-10 SNPs revealed no differences between patients and controls, suggesting the absence of influence of both SNPs on SLE susceptibility (Table 2). However, a significant effect of the interaction between IL-10 rs1800896 and Atg5 rs573775 was observed in the multivariate analysis (Table 3). Thus, to further specify the way of this interaction, we analyzed the influence of Atg5 rs573775 on SLE risk in genetically high (GG/GA) and low (AA) IL-10 producers. After grouping by IL-10 genotype (Table 4), we observed that presence of the Atg5 T* minor allele was a risk factor for SLE in carriers of the high IL-10 producer genotype, but not among low IL-10 producers, where it seemed to exert the opposite effect. Accordingly, the presence of the high IL-10 producer allele was a risk factor for SLE in carriers of the Atg5 mutation (OR, 95% CI: 2.19, 1.05-4.57; p=0.04) but not in Atg5 wild type individuals (OR, 95% CI: 0.54, 0.27-1.1; p=0.09). Therefore, association of Atg5 rs573775 SNP on SLE susceptibility could be dependent on other factors, such as the IL-10 genotype.

**Table 2:** Genotypic and allelic distributions of Atg5 and IL-10 SNPs in controls and SLE patients.

	**Controls**		**SLE**		
	**n (%)**		**n (%)**		**p-value**
**Atg5 rs573775**					
CC	85 (50.00)		56 (48.70)		0.725
CT	71 (41.76)		52 (45.22)		
TT	14 (8.24)		7 (6.08)		
T* allele frequency	29.12%		28.94%		0.913
**IL-10 rs1800896**					
AA	65 (38.23)		42 (36.52)		0.447
AG	78 (45.88)		48 (41.74)		
GG	27 (15.87)		25 (21.73)		
G* allele frequency	38.82%		42.61%		0.366

Differences were evaluated by χ^2^ test.

**Table 3:** Atg5 and IL-10 SNPs interaction on SLE susceptibility.

	**Wald value**	**p-value**
Atg5 rs573775	3.06	0.216
IL-10 rs1800896	2.89	0.235
Atg5 rs573775 * IL-10 rs1800896	10.39	0.034

Associations were evaluated by multivariate logistic regression adjusted for sex and age. The accuracy of the model was 64.50%.

**Table 4:** Association of Atg5 rs573775 alleles on SLE susceptibility in genetically high and low IL-10 producers.

	**IL-10 high (GG/AG)**						**IL-10 low (AA)**				
	**Controls**		**SLE patients**				**Controls**		**SLE patients**		
	**n** (**%**)		**n (%)**	**OR (95% CI)**	**p-value**		**n (%)**		**n (%)**	**OR (95% CI)**	**p-value**
**Atg5 wild (CC)**	58 (34.1)		30 (26.1)	Reference			27 (15.9)		26 (22.6)	Reference	
**Atg5 mutated (CT/TT)**	47 (27.6)		43 (37.4)	1.912 (1.00-3.575)	**0.042**		38 (22.4)		16 (13.9)	0.468 (0.203-1.079)	0.075

Calculated by unconditional logistic regression modeling adjusted for sex and age. The accuracy of the models was 61.10% for IL-10 high and 60.70 % for IL-10 low.CI, confidence interval; OR, odds ratio.

### 3. Atg5/IL-10 combined genotype influences SLE features

Next, in an attempt to determine the possible role of IL-10/Atg5 genotypes in the phenotypic manifestations of lupus disease, SLE patients were classified into four IL-10/Atg5 combined genotypes and both cytokine serum levels and clinical features were evaluated. 

Table 5 shows that carriers of the mutated Atg5 T* allele presented significantly lower amounts of IFNα, while TNFα presented the same trend but was not statistically significantly, when compared with the other patients. Serum levels of IL-10, however, were only significantly increased in patients with mutated Atg5 and genetically high IL-10 producers. To determine the strength of the association between circulating cytokines and the studied genetic variants, a quantitative model was performed for each cytokine (Table 6). According to previous data (Figure 1), Atg5 rs573775 significantly influences, but in an opposite way, IFNα and IL-10 levels, whereas a clear trend was detected for TNFα. Conversely, IL-10 rs1800896 only showed a significant influence on IFNα levels. However, we did not find significant effect of the rs573775 - rs1800896 interaction on such cytokine serum levels. On the other hand, the analysis of clinical characteristics (Table 7) showed that high IL-10 producers carriers of the Atg5 T* allele presented significantly lower prevalence of cytopenia and a trend of less renal disorder compared with the other genotypes. However, patients with the Atg5 mutated allele and carriers of the low IL-10 producer genotype were oldest at diagnosis and presented a clear trend toward a lower frequency of anti-dsDNA autoantibodies. 

**Table 5:** Cytokine serum levels in SLE patients with different IL-10/Atg5 genotype.

**Combined genotype**	**IFNα (pg/ml)**	**TNFα (pg/ml)**	**IL-10 (pg/ml)**
IL-10 high / Atg5 mutated	17.13 (35.19)^a^	31.01 (83.86)^c^	1.40 (2.89)^e^
IL-10 low / Atg5 mutated	15.50 (46.75)^a^	32.29 (97.65)^c^	0.41 (1.83)
IL-10 high / Atg5 wild	46.58 (241.95)^b^	60.98 (113.92)^d^	0.20 (0.84)
IL-10 low / Atg5 wild	23.19 (95.00)^b^	88.15 (169.39)^d^	0.20 (2.28)

Values are median (interquartile range). Differences were evaluated by Mann-Whitney U test between the following groups:

**a** *vs* **b**: p=0.010

**c** *vs* **d**: p=0.130

**e** *vs* **others**: p=0.022

**Table 6:** Influence of Atg5 and IL-10 SNPs on cytokine serum levels in SLE patients.

	**IFNα**			**TNFα**			**IL-10**	
	Standardized linear regression coefficient	p-value		Standardized linear regression coefficient	p-value		Standardized linear regression coefficient	p-value
Atg5 rs573775	-0.271	**0.007**		-0.186	0.052		0.252	**0.015**
IL-10 rs1800896	0.212	**0.033**		0.133	0.163		-0.014	0.894
Sex	0.022	0.825		-0.003	0.973		-0.033	0.746
Age	-0.178	0.072		-0.003	0.977		-0.034	0.740

Differences were evaluated by multivariate linear regression analysis using IFNα, TNFα or IL-10 levels as the dependent variable and adjusted for age and sex.

**Table 7:** Clinical features of SLE patients according to IL-10/Atg5 combined genotype.

	**IL-10 high / Atg5 mutated**	**IL-10 low / Atg5 mutated**	**IL-10 high / Atg5 wild**	**IL-10 low / Atg5 wild**
	**n=43**	**n=16**	**n=30**	**n=26**
**Age at diagnosis, years (mean±SEM)**	30.21 (0.37)	36.69 (0.58)^3^	29.33 (0.44)	28.04 (0.46)
**Clinical manifestations, n (%**)				
Malar rash	22 (52.16)	10 (62.50)	19 (63.33)	17 (65.38)
Discoid lesions	9 (20.93)	3 (18.75)	4 (13.33)	7 (26.92)
Photosensitivity	23 (53.49)	10 (62.50)	15 (50.00)	17 (65.38)
Oral ulcers	13 (30.23)	7 (43.75)	12 (40.00)	14 (53.85)
Arthritis	33 (76.74)	11 (68.75)	25 (83.33)	17 (65.38)
Serositis	11 (25.58)	2 (12.50)	5 (16.67)	7 (26.92)
Cytopenia	16 (38.09)^1^	10 (62.50)	18 (60.00)	17 (65.38)
Renal disorder	9 (20.93)^2^	6 (37.50)	9 (30.00)	12 (46.15)
Neurological disorder	4 (9.30)	2 (12.50)	0 (0.00)	2 (7.69)
Anti-dsDNA	32 (74.42)	9 (56.25)^4^	24 (80.00)	20 (76.92)

Differences were evaluated by the χ^2^ test (categorical variables) or the Mann Whitney U test (continuous variables) between patients carriers of the indicated genotype and the rest ^1^:p= 0.012 ^2^,p= 0.064 ^3^,p= 0.017 ^4^,p= 0.083.

**Figure 2:**
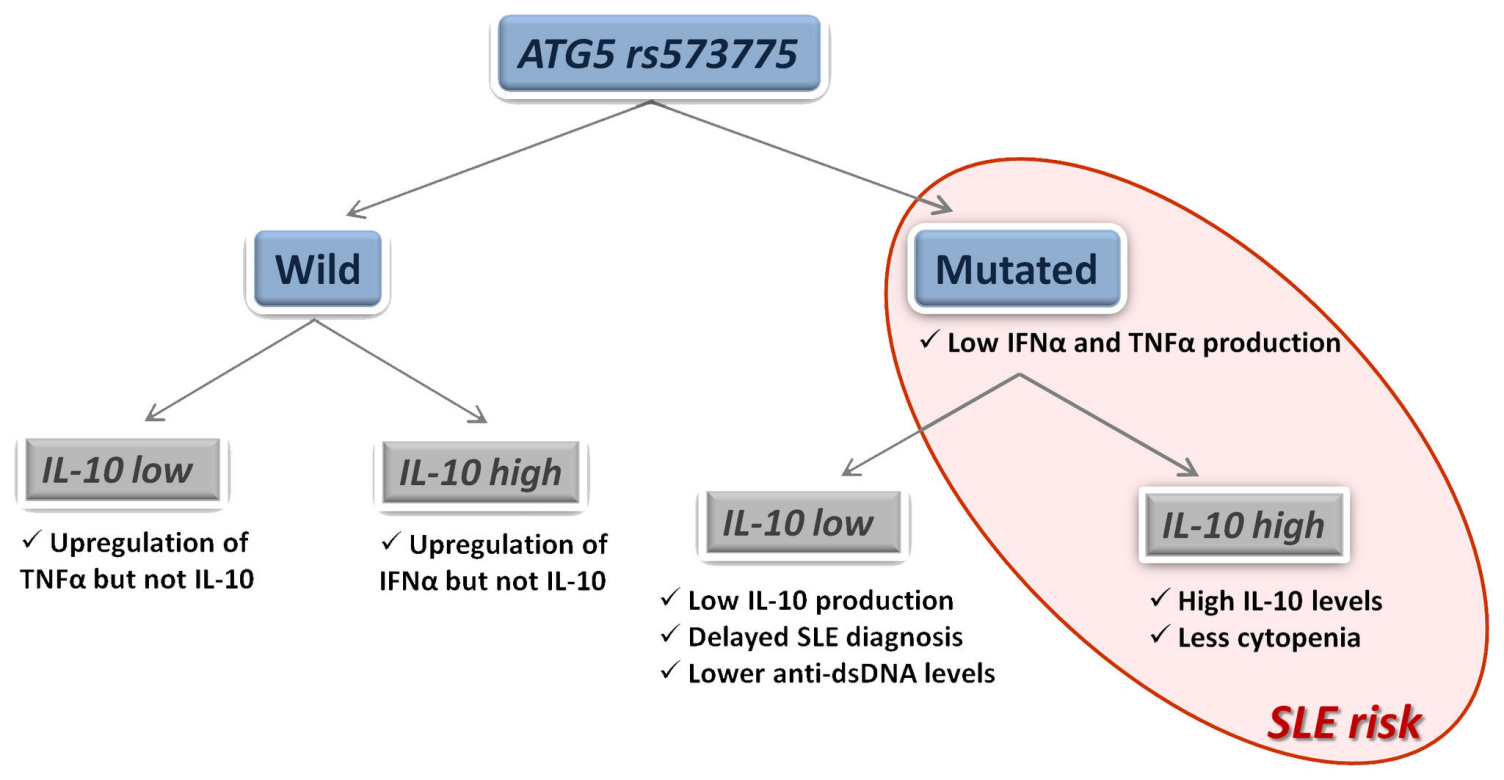
Effects of Atg5 and IL-10 SNPs on SLE risk, cytokine levels and clinical features. The diagram represents a model of the effects of Atg5 rs573775 and the functional IL-10 polymorphisms (low/high genetic producers) on SLE, which were supported by our results. A significant influence of both SNPs was detected, however, Atg5 rs573775 showed a dominant role.

Therefore, the presence of the Atg5 mutated allele seems to be associated with specific clinical and immunological features in patients with SLE, but differs depending on the functional IL-10 genotype (Figure 2). Thus, presence of the Atg5 T* allele in combination with the high IL-10 genotype was associated with the highest IL-10 levels, reduced production of IFNα and TNFα and the lowest frequency of hematological and renal disorder, whereas carriers of the same mutation but in the presence of the low IL-10 genotype showed lower levels of all the studied cytokines, less frequency of anti-dsDNA antibodies and delayed onset of the disease, in accordance with the lower frequency of this genotype in SLE patients. 

## Discussion

Alterations in autophagy and in several Atg regulatory proteins are supposed to have a role in the etiology of autoimmune disorders [25]. However, although genome-wide association studies showed that various SNPs in the Atg5 locus were associated with SLE susceptibility [21–24], the possible effects of such genetic variations in SLE patients are currently unknown. 

In the present work we describe, for the first time, an interesting relationship between an Atg5 SNP and cytokine serum levels of SLE patients. Specifically, carriers of the rs573775 T* minor allele, previously associated with SLE susceptibility in two large cohorts [21,26], presented lower IFNα and higher IL-10 levels than patient carriers of the wild genotype. In addition, a slight trend towards lower production of TNFα, a cytokine positively correlated with IFNα in SLE patients was also observed. In accordance with these results, it has been reported that disruption of basal autophagy can reduce the induction of type I IFN in murine pDCs [35] and the production of TNFα by human peripheral blood mononuclear cells [36]. Since an increased production of IFNα, probably by pDC, is a key feature in the etiopathogenesis of SLE, this association between a putative SLE risk factor and low IFNα levels might seem contradictory. Some evidence, however, suggests it is consistent. It has been reported that the autophagy process participates in the induction of type I IFNs in pDC upon binding of nucleic acids to the TLR7/9 [35]. Moreover, the most pronounced defect of mice with DC-conditional deletion in Atg5 was the processing and presentation of phagocytosed antigens containing TLR stimuli for MHC class II, then failing to mount a proper Th1 cell immune response [13]. In addition, Atg5 could play a role in non-canonical autophagy that mediates IFNα secretion in response to DNA-immune complexes [37,38]. Therefore, Atg5-mediated alterations in autophagy may impair the activation of DCs through the immune complexes present in SLE patients, thus preventing the production IFNα and the activation of NF-κB, a pathway involved in the transcription of Th1-promoting cytokines, TNFα and other proinflammatory mediators. 

On the other hand, our results might suggest that Atg5 mutation in SLE patients prompts the upregulation of IL-10, usually increased in these patients and associated with indicators of disease activity [39]. This cytokine promotes B-cell-mediated functions, enhancing survival, proliferation, differentiation and antibody production, thus explaining B cell hyperactivity and autoantibody production, two main features of SLE. It is known that IL-10 has a predominant opposite effect to TNFα in systemic inflammatory responses, both cytokines being mutually regulated in physiological conditions. It is worth noting that functional IL-10 SNPs, especially rs1800896, have been associated in several populations with SLE susceptibility or clinical outcome [33], although conclusive data have not been obtained. Thus, at this point, it seems reasonable to think that IL-10 and Atg5 SNPs could exert a combined effect on SLE disease. 

With regard to the association of IL-10 rs1800896 and Atg5 rs573775 SNPs to SLE susceptibility, no significant differences in allelic or genotypic frequencies were observed in our cohort between patients and controls. The IL-10 gene is situated in a major SLE susceptibility locus (1q31-32), however, in spite of the considerable number of studies performed, no definitive result about its involvement on SLE susceptibility was achieved [33], which could be due, at least in part, to the differences in the frequencies of the IL-10 rs1800896 alleles among populations. In a similar way, the studies on the involvement of several Atg5 SNPs showed inconclusive results, although the Atg5 rs573775 T* allele has been found significantly associated with SLE susceptibility, at a low rate (OR: 1.17 and 1.19), in two large cohorts that included different populations [21,26]. Our results could shed some light on this matter, since we observed that the association of Atg5 SNP with SLE susceptibility may be dependent on other factors, such as the IL-10 genotype. The present work indicates that the Atg5 rs573775 T* allele was a risk factor for SLE in carriers of the IL-10 rs1800896 G* high producer allele, but not among genetically low IL-10 producers. Interestingly, in our Spanish population, like in other South European countries, the IL-10 G* allele was underrepresented compared with North/Central European and North American populations [33]. These differences could explain the lack of a significant association of the Atg5 rs573775 SNP with susceptibility when analyzing our entire population, in which about 60% of individuals are high IL-10 producers whereas in North/Central Europeans they may represent 75%. It is of note, Alonso-Perez et al. [26] showed that this Atg5 SNP was more associated with SLE susceptibility in Central than in Southern Europeans (OR, 95% CI: 1.36, 1.10-1.68 and 1.11, 0.98-1.26 , respectively), in accordance with the frequency of genetically high IL-10 producers reported in these populations.

Hence, in spite of the lack of IFNα induction, the Atg5 mutation was a risk factor for SLE when high IL-10 levels are available. In line with this, our results showed that genetically high IL-10 producer patients presented high serum levels of this cytokine when the Atg5 mutated allele was present, but not in Atg5 wild type individuals. Accordingly, the linear multivariate regression model performed in this work suggests that Atg5 mutation, rather than IL-10 SNP, exerts a relevant role in the prediction of IL-10 serum levels. Therefore, cytokine levels of SLE patients classified in the four combined IL-10/Atg5 genotypes suggested that those with normal autophagy function (Atg5 wild type), regardless of their IL-10 genotype, produced enough TNFα to counterbalance IL-10 levels, whereas Atg5 mutated patients were unable to control the genetically high IL-10 production (Table 5). In addition, it has been reported that IL-10 and other Th2 cytokines may inhibit the induction of autophagy in several immune cells [40,41], thus enhancing the effect of Atg5 mutation on SLE susceptibility.

Finally, the combined IL-10/Atg5 genotypes also showed an impact on the clinical characteristics of lupus disease. Intriguingly, IL-10 genotype seems to influence the outcome of SLE patients who are carriers of the Atg5 mutated allele. Thus, high IL-10 producers mutated for Atg5 exhibited the lowest frequency of cytopenia, suggesting an effect of this cytokine in increasing the survival of activated lymphocytes. In this sense, since autophagy is able to regulate cell death in activated T cells [42,43], the presence of an altered Atg5 function in patients carriers of the mutated allele could favor the maintenance and activation of autoreactive lymphocytes in SLE. On the other hand, low IL-10 producer patients mutated for Atg5 displayed the lowest amount of anti-dsDNA antibodies and later onset of the disease. These features could be explained by the low serum levels of IL-10, IFNα, TNFα, and probably other cytokines, together with the altered autophagy function present in patients with this genotype, which were slightly underrepresented in our population compared with controls. 

In conclusion, this is the first study carried out in SLE patients that has analyzed the influence of functional IL-10 genotype on the effect of Atg5 mutation, two molecules previously associated with susceptibility or outcome of the disease. We found that Atg5 mutation was a risk factor for SLE in carriers of the high IL-10 producer allele, leading to high IL-10 and low IFNα production and low frequency of cytopenia. Contrary, presence of this Atg5 mutation in low IL-10 producers did not increase SLE risk, patients with this genotype exhibiting low amounts of the analyzed cytokines, the lowest prevalence of anti-dsDNA antibodies and a delay in the age at diagnosis (Figure 2).
